# Cost-Effectiveness Analysis of Sofosbuvir Compared to Current Standard Treatment in Swiss Patients with Chronic Hepatitis C

**DOI:** 10.1371/journal.pone.0126984

**Published:** 2015-05-14

**Authors:** Alena M. Pfeil, Oliver Reich, Ines M. Guerra, Sandrine Cure, Francesco Negro, Beat Müllhaupt, Daniel Lavanchy, Matthias Schwenkglenks

**Affiliations:** 1 Institute of Pharmaceutical Medicine (ECPM), University of Basel, Basel, Switzerland; 2 Department of Health Sciences, Helsana Group, Zurich, Switzerland; 3 OPTUMInsight, Uxbridge, Middlesex, United Kingdom; 4 Divisions of Gastroenterology and Hepatology and of Clinical Pathology, University Hospital Geneva, Geneva, Switzerland; 5 Swiss HPB (Hepato-Pancreato-Biliary) Center and Department of Gastroenterology and Hepatology, University Hospital Zürich, Zurich, Switzerland; 6 World Health Organization (WHO), African Union, Governments, Denges VD, Switzerland; National Taiwan University Hospital, TAIWAN

## Abstract

In clinical trials, sofosbuvir showed high antiviral activity in patients infected with hepatitis C virus (HCV) across all genotypes. We aimed to determine the cost-effectiveness of sofosbuvir-based treatment compared to current standard treatment in mono-infected patients with chronic hepatitis C (CHC) genotypes 1–4 in Switzerland. Cost-effectiveness was modelled from the perspective of the Swiss health care system using a lifetime Markov model. Incremental cost-effectiveness ratios (ICERs) used an endpoint of cost per quality-adjusted life year (QALY) gained. Treatment characteristics, quality of life, and transition probabilities were obtained from published literature. Country-specific model inputs such as patient characteristics, mortality and costs were obtained from Swiss sources. We performed extensive sensitivity analyses. Costs and effects were discounted at 3% (range: 0–5%) per year. Sofosbuvir-containing treatment in mixed cohorts of cirrhotic and non-cirrhotic patients with CHC genotypes 1–4 showed ICERs between CHF 10,337 and CHF 91,570 per QALY gained. In subgroup analyses, sofosbuvir dominated telaprevir- and boceprevir-containing treatment in treatment-naïve genotype 1 cirrhotic patients. ICERs of sofosbuvir were above CHF 100,000 per QALY in treatment-naïve, interferon eligible, non-cirrhotic patients infected with genotypes 2 or 3. In deterministic and probabilistic sensitivity analyses, results were generally robust. From a Swiss health care system perspective, treatment of mixed cohorts of cirrhotic and non-cirrhotic patients with CHC genotypes 1–4 with sofosbuvir-containing treatment versus standard treatment would be cost-effective if a threshold of CHF 100,000 per QALY was assumed.

## Introduction

Hepatitis C virus (HCV) is a ribonucleic acid (RNA) virus causing acute and chronic hepatitis [1]. Worldwide, the HCV prevalence is about 3% [2]. In Europe and the US, HCV infection through injection drug use has become the major transmission route [3]. Although most patients infected with HCV are symptomless, chronic hepatitis C (CHC) poses a significant risk of developing cirrhosis and hepatocellular carcinoma, if left untreated [4]. Thus, CHC is a cause of major health burden causing substantial morbidity and mortality [5]. In the US, costs of about 6.5 billion per year are estimated [6] despite the availability of antiviral therapy.

The aim of therapy in chronic hepatitis C is to achieve a sustained virological response (SVR). SVR is defined as undetectable serum HCV RNA after the end of treatment, signalling eradication of HCV infection [7]. SVR at 12 weeks has shown high concordance with SVR at 24 weeks [7] and has been accepted by regulators in the US and Europe as an appropriate endpoint indicating treatment success [8]. Response to HCV treatment differs according to HCV genotype, disease stage, and HCV treatment history [9]. Pegylated interferon alpha and ribavirin have long been considered standard of care [8] with SVR rates of 40–50% in genotypes 1 and 4 [10] and SVR rates up to 80% in patients with genotypes 2 and 3 [11]. Due to significant side effects and contraindications associated with pegylated interferon alpha and ribavirin therapy, direct-acting antivirals have been developed [9]. Protease inhibitors such as telaprevir and boceprevir have been licensed since 2011 for HCV genotype 1 and have increased SVR rates, but major safety and efficacy issues persist [9].

Sofosbuvir, a newly developed uridine nucleotide analogue HCV NS5B polymerase inhibitor, has shown high antiviral activity across genotypes and few severe side-effects in a range of clinical trials including various patient populations [12–16]. In treatment-naïve genotype 1 patients, triple therapy with sofosbuvir, pegylated interferon alpha and ribavirin for 12 weeks reached a SVR of 89% in a phase III trial [13]. SVR was 96% and 83% in a phase II trial enrolling treatment-experienced patients with genotypes 2 and 3 receiving 12 weeks of triple therapy with sofosbuvir, pegylated interferon alpha and ribavirin [16]. Treatment with sofosbuvir in combination with pegylated interferon alpha and/or ribavirin recently received approval for reimbursement by the Swiss statutory health insurance in patients with CHC and fibrosis stage 3 or 4, or symptomatic patients with extra hepatic manifestations [17].

The aim of this cost-effectiveness analysis was to estimate clinical effectiveness in terms of quality-adjusted life years (QALYs) gained, the direct medical cost, and the cost-effectiveness in terms of cost per QALY gained, of sofosbuvir-based treatment strategies compared with the current standard treatment of mono-infected patients with CHC genotypes 1–4. The article follows the CHEERS statement for reporting health economic evaluations [18].

## Materials and Methods

### Patient population and treatment strategies

We evaluated non-HIV-infected patients diagnosed with CHC genotypes 1–4. Patient groups were further subdivided into treatment-naïve *versus* treatment-experienced and interferon-eligible patients *versus* patients unsuitable for interferon (interferon-ineligible patients or patients unwilling to take interferon). To reflect real-life medical practice, we obtained average Swiss CHC patient characteristics, such as mean age, mean weight and percentage of cirrhotic patients by genotype, from the Swiss Hepatitis C Cohort Study (SCCS) [19]. This representative cohort study collects standardised prospective data on demographics, laboratory markers, HIV infection status, treatment and treatment results of HCV infected patients in Switzerland aged 18 years and over, and is fairly representative of the overall infected population in terms of age, sex distribution and risk factors for HCV acquisition [19]. The following data points were extracted from the SCCS in May 2014. The percentage of cirrhotic patients was 24% in genotype 1, 21% in genotype 2, 25% in genotype 3 and 22% in genotype 4. Mean age was 54, 53 and 51 years and mean body weight 73.5, 73.6 and 73.7 kilograms for genotype 1, genotypes 2 and 3, and genotype 4, respectively. The percentage of cirrhotic patients obtained from the SCCS was used for the base-case analysis.

Pairs of treatment strategies containing sofosbuvir and comparator strategies representing current standard of care were identified by experienced Swiss clinicians, based on their relevance for Switzerland and taking into account the local prevalence of different genotypes. No treatment was included as the comparator for CHC patients unsuitable for interferon treatment, in the absence of treatment alternatives. Treatment regimens were implemented in the model as per their marketing authorisations and according to the European Association for the Study of the Liver (EASL) guidelines [20].

### Health economic model characteristics

**Fig 1:**
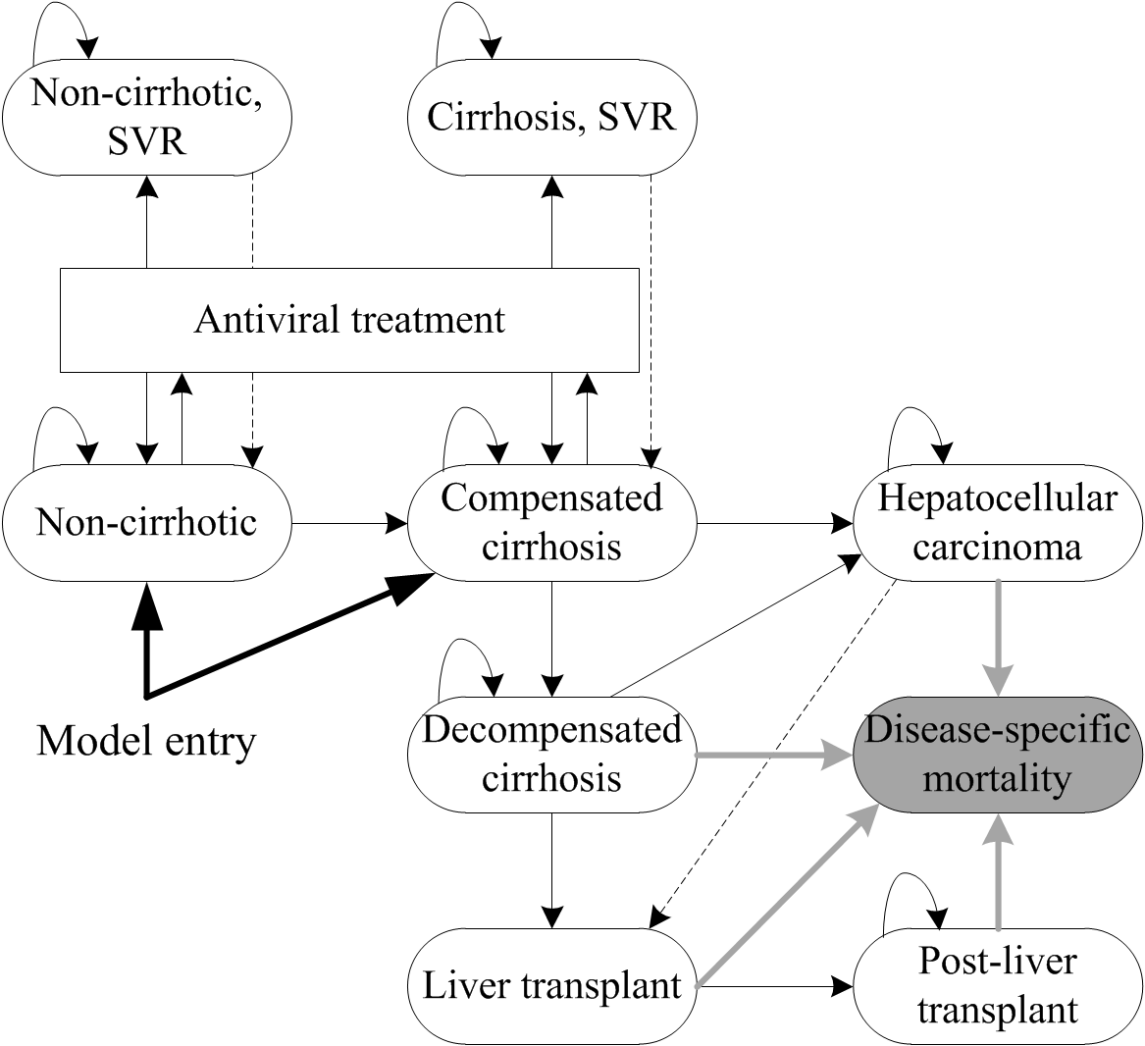
Structure of the Markov state transition model. Patients can transition from each health state to death from any cause.

We used a Markov state-transition model with a life-long time horizon implemented in MS Excel; the structure is shown in Fig 1. The model structure of two health economic models developed by the Southampton Health Technology Assessment Centre [21,22] that were submitted to the UK National Institute for Health and Care Excellence (NICE) served as basis and was adapted to achieve reconciliation with available data from clinical trials. Specifically, mild (METAVIR [23] score F0-F1, absent or portal fibrosis) and moderate HCV (METAVIR score F2 or F3, portal fibrosis with few or several septa) states included in the original models were combined to a non-cirrhotic state to reflect available data from clinical trials. In the resulting structure, virtual mixed cohorts of 10,000 patients with no cirrhosis or compensated cirrhosis entered the model at the beginning of CHC treatment. Possible health states included non-cirrhotic with SVR, cirrhosis with SVR, decompensated cirrhosis, hepatocellular carcinoma, liver transplant, post-liver transplant and death (Fig 1). Disease-specific mortality (excess mortality) and general mortality were included separately; patients were exposed to age-specific probabilities of death in the general population [24] in each health state. The possibility of recurrences and relapses were tested in sensitivity analysis (dotted arrow in Fig 1). The disease was assumed not to progress while patients were on treatment and 12 weeks after the end of treatment. Patients could not die during treatment. A half-cycle correction was used for costs as well as effects. Costs (Swiss Francs, CHF 2014) and effects were discounted at 3% (range in sensitivity analysis: 0–5%) as recommended in current literature [25]. We calculated the cost-effectiveness of sofosbuvir in terms of cost per quality-adjusted life year (QALY) from the perspective of the Swiss health care system (indirect costs not included). There is no published or generally accepted cost-effectiveness threshold for Switzerland but other Swiss cost-effectiveness studies have used CHF 100,000 per QALY gained to distinguish potentially favourable from unfavourable incremental cost-effectiveness ratios (ICERs) [26–28].

### Model input parameters and assumptions

Transition probabilities, quality of life weights, treatment efficacy and safety data, resource use data, and treatment-related adverse event (AE) costs were obtained from published clinical trial reports, other peer-reviewed literature and treatment guidelines. Patient characteristics, all-cause mortality, treatment-related costs and health state costs were extracted from Swiss-specific sources.

Annual transition probabilities between health states were extracted from published literature as referenced in Table 1. Probabilities of death in the general population were obtained from the Swiss Federal Statistical Office [24].

**Table 1:** Base-case values for model input parameters and ranges used in the sensitivity analyses.

	Item		Source	Base-case		DSA ranges		PSA distribution and parameters	
*Annual transition probabilities*	From	To		GT1	others	Lower -25%	Upper +25%	GT1	others
	Non-cirrhotic 30 years	Compensated cirrhosis	[30], [31]	0.006	0.009	0.0015	0.0105	Beta, α = 9 β = 1481	Beta, α = 20 β = 2209
	Non-cirrhotic 40 years	Compensated cirrhosis	[30], [31]	0.010	0.014	0.0025	0.0175	Beta, α = 11 β = 1088	Beta, α = 21 β = 1511
	Non-cirrhotic 50 years	Compensated cirrhosis	[30], [31]	0.016	0.025	0.0048	0.0280	Beta, α = 10 β = 619	Beta, α = 8 β = 316
	Non-cirrhotic, SVR	Recurrence	Expert opinion	Not assessed in base-case	Not assessed in base-case	0–0.01	0–0.01	-	-
	Non-cirrhotic, SVR	Re-infection	Expert opinion	Not assessed in base-case	Not assessed in base-case	0–0.01	0–0.01	-	-
	Compensated cirrhosis	Decompensated cirrhosis	[29]	0.039	0.039	0.0219	0.0608	Beta, α = 15 β = 360	Beta, α = 15 β = 360
	Compensated cirrhosis	Hepatocellular carcinoma	[21,22,29]	0.014	0.014	0.0016	0.0392	Beta, α = 2 β = 136	Beta, α = 2 β = 136
	Compensated cirrhosis, SVR	Recurrence	Expert opinion	Not assessed in base-case	Not assessed in base-case	0–0.01	0–0.01	-	-
	Compensated cirrhosis, SVR	Re-infection	Expert opinion	Not assessed in base-case	Not assessed in base-case	0–0.01	0–0.01	-	-
	Decompensated cirrhosis	Hepatocellular carcinoma	[21,22,29]	0.014	0.014	0.002	0.039	Beta, α = 2 β = 136	Beta, α = 2 β = 136
	Decompensated cirrhosis	Liver transplant	[22]	0.030	0.030	0.012	0.056	Beta, α = 7 β = 211	Beta, α = 7 β = 211
	Decompensated cirrhosis	Death	[21,22,29]	0.130	0.130	0.111	0.150	Beta, α = 147 β = 984	Beta, α = 147 β = 984
	Hepatocellular carcinoma	Liver transplant	Expert opinion	0–0.01	0–0.01	-	-	-	-
	Hepatocellular carcinoma	Death	[21,22,29]	0.43	0.43	0.37	0.49	Beta, α = 117 β = 155	Beta, α = 117 β = 155
	Liver transplant	Death, year 1	[22]	0.21	0.21	0.13	0.31	Beta, α = 16 β = 61	Beta, α = 16 β = 61
	Post-liver transplant	Death, year 2	[22]	0.057	0.057	0.037	0.082	Beta, α = 23 β = 379	Beta, α = 23 β = 379
Probability of death for the general population in 2003*	Age-group	Annual	3-month	Source	Base-case	Lower -25%	Upper +25%	PSA	PSA
	15–24	0.000522	0.000130	SFSO	0.000522	0.0004	0.0007	-	-
	25–34	0.000660	0.000165	SFSO	0.000660	0.0005	0.0008	-	-
	35–44	0.001128	0.000282	SFSO	0.001128	0.0008	0.0014	-	-
	45–54	0.002758	0.000689	SFSO	0.002758	0.0021	0.0034	-	-
	55–64	0.006848	0.001711	SFSO	0.006848	0.0051	0.0086	-	-
	65–74	0.017697	0.004145	SFSO	0.017697	0.0133	0.0221	-	-
	77–84	0.054529	0.013540	SFSO	0.054529	0.0409	0.0682	-	-
	85+	0.165443	0.040517	SFSO	0.165443	0.1241	0.2068	-	-
*Quality of life—utilities*	Health state			Source	Base-case	Lower -25%	Upper +25%	PSA	PSA
	Non-cirrhotic			[29]	0.74	0.71	0.77	Beta, α = 707 β = 248	Beta, α = 707 β = 248
	Compensated cirrhosis			[29]	0.55	0.44	0.65	Beta, α = 47 β = 39	Beta, α = 47 β = 39
	Patients on treatment with SOF (utility decrement)			[13]	-0.15	-0.20	+0.20	Gamma, α = 63 β = 0.002	Gamma, α = 63 β = 0.002
	Patients receiving comparator (utility decrement)			[13]	-0.15	-0.12	-0.18	Gamma, α = 8 β = 0.02 (PegIFN+RBV)	Gamma, α = 8 β = 0.02 (PegIFN+RBV)
	Patients receiving comparator (utility decrement)			[13]	-0.15	-0.12	-0.18	Gamma, α = 204 β = 0.0007 (Telaprevir)	Gamma, α = 204 β = 0.0007 (Telaprevir)
	Patients receiving comparator (utility decrement)			[13]	-0.15	-0.12	-0.18	Gamma, α = 143 β = 0.0009 (Boceprevir)	Gamma, 03B1 = 143 β = 0.0009 (Boceprevir)
	SVR (utility increment)			[29]	+0.05	+0.002	+0.17	Gamma, α = 1.25 β = 0.04	Gamma, α = 1.25 β = 0.04
	Non-cirrhotic, SVR			0.74+0.05	0.79	-	-	-	-
	Cirrhotic, SVR			0.55+0.05	0.60	-	-	-	-
	Decompensated cirrhosis			[29]	0.45	0.39	0.51	Beta, α = 124 β = 151	Beta, α = 124 β = 151
	Hepatocellular carcinoma			[29]	0.45	0.39	0.51	Beta, α = 124 β = 151	Beta, α = 124 β = 151
	Liver transplant			[29]	0.45	0.39	0.51	Beta, α = 124 β = 151	Beta, α = 124 β = 151
	Post-liver transplant			[29]	0.67	0.61	0.73	Beta, α = 163 β = 80	Beta, α = 163 β = 80

DSA, deterministic sensitivity analysis; GT, genotype; HCC, hepatocellular carcinoma; PegIFN, pegylated interferon; PSA, probabilistic sensitivity analysis; RBV, ribavirin; SFSO, Swiss Federal Statistical Office; SOF, Sofosbuvir; SVR, sustained virological response

*Obtained by converting mortality rates using the formulae probability = 1-exp(-rate*time)

Treatment duration, efficacy and safety were obtained from randomised and non-randomised phase II and phase III clinical trials as presented and referenced in Table 2. The percentages of patients and the time point at which these patients have discontinued treatment due to adverse events (AEs) or other reasons before the end of the planned treatment duration are provided in Table 2. Incidences of adverse events (AEs) such as anaemia, nausea, vomiting and rash are provided in S1 Table. Efficacy data were obtained from phase II trials if the corresponding phase III trials had missing data, e.g. did not report SVR rates according to cirrhosis status, or the treatment strategy did not match, e.g. SVR rates were reported for 12 instead of 24 weeks of treatment. There was one head-to-head trial available in genotypes 2 and 3 patients which compared a combination of sofosbuvir and ribavirin with a regimen consisting of pegylated interferon alpha and ribavirin [13]. Other efficacy and safety data were obtained from available randomised trials and single-arm studies with comparable patient characteristics.

**Table 2:** Genotype-specific parameters including treatment efficacy, duration and safety.

Indication [Source]	SOF-based and comparator strategies	SVR		Discontinued#		Patients with adverse events	
		cirrhotic	Non-cirrhotic	Due to AEs	Other reasons	Any	Serious
GT1 TN IE [13,32,33]	SOF + PegIFN2a + RBV for 12 wks	80.8%	91.3%	1.7% at 5.3 wks	0.7% at 4.8 wks	95%	1%
	PegIFN2a/2b + RBV for 48 wks	23.6%	43.6%	7.0% at 24 wks	24% at 24 wks	96%	1%
	TEL + PegIFN2a + RBV for 24/48 wks	61.9%	75.4%	NA	23.3% at 18 wks	100%	21%
	BOC + PegIFN2b + RBV for 28/48 wks	55.0%	64.1%	NA	26.1% at 24 wks	99%	13%
GT1 TN II [34]	SOF + RBV for 24 wks	53.3%	68.3%	1% at 12 wks	0%	89%	0%
	NT	0%	0%	0%	0%	69%	0%
GT2 TN IE [13]*	SOF + RBV for 12 wks	90.9%	98.3%	0%	0%	86%	3%
	PegIFN2a/2b + RBV for 24 wks	61.5%	81.5%	12% at 14.9 wks	6	96%	1%
GT2 TN II [12]	SOF + RBV for 12 wks	93.3%	91.8%	1% at 0.9 wks	1% at 1.3 wks	89%	5%
	NT	0%	0%	0%	0%	78%	0%
GT3 TN IE [13,16,35,36]	SOF + RBV for 24 wks	92.3%	93.5%	0.4% at 21.5 wks	1.2% at 21.5 wks	92%	4%
	SOF + PegIFN2a + RBV for 12 wks	83.3%	100%	0%	0%	NA	1%
	PegIFN2a/2b + RBV for 24 wks	29.7%	71.2%	10.2% at 10.8 wks	13.6% at 11.9 wks	96%	1%
GT3 TN II [35]	SOF + RBV for 24 wks	92.3%	93.5%	0.4% at 21.5 wks	1.2% at 21.5 wks	92%	4%
	NT	0%	0%	0%	0%	71%	0%
GT3 TE IE [16,35,37,38]	SOF + RBV for 24 wks	62%	87%	0.4% at 21.5 wks	1.2% at 21.5 wks	92%	4%
	SOF + PegIFN2a + RBV for 12 wks	83.3%	83.3%	8% at 1 wk	0%	96%	1%
	PegIFN2a/2b + RBV for 48 wks	35%	35%	36.8% at 24 wks	0%	100%	1%
GT3 TE II [35]	SOF + RBV for 24 wks	60%	85%	0.4% at 21.5 wks	1.2% at 21.5 wks	92%	4%
	NT	0%	0%	0%	0%	71%	0%
GT4 TN [13,39]	SOF + PegIFN2a + RBV for 12 wks	50%	100%	0%	0%	95%	2%
	PegIFN2a/2b + RBV for 48 wks	38.6%	50%	14% at 24 wks	26% at 24 wks	96%	1%

BOC, boceprevir; GT, genotype; IE, interferon eligible; II, interferon ineligible; NA, not available; PegIFN, pegylated interferon; RBV, ribavirin; SOF, sofosbuvir; SVR, sustained virological response; TE, treatment-experienced; TEL, telaprevir; TN, treatment-naïve

* head-to-head comparison trial

# the percentages of patients and the time point at which these patients have discontinued treatment due to adverse events or other reasons before the end of the planned treatment duration

Quality of life weights (utilities) for the health states represented in the model, based on the European quality of life questionnaire (EQ-5D), were obtained from published literature (Table 1). For patients on treatment with sofosbuvir or a comparator, a utility decrement obtained in a sofosbuvir trial based on the short-form health survey (SF-6D) was applied [13]. When non-cirrhotic or cirrhotic patients reached SVR after treatment, utilities obtained in a UK randomised controlled trial of interferon and ribavirin based on the EQ-5D [29] were added to the utility of the corresponding health state (Table 1).

Resource use parameters such as tests and procedures related to CHC diagnosis and treatment were obtained from EASL Clinical Practice Guidelines [8,20] (S2 Table). Swiss clinical experts checked whether they reflected Swiss clinical practice. Experts were selected to represent all Swiss regions and university hospitals. In Swiss regions without a university hospital, clinical experts from hospitals experienced in the treatment of HCV patients were included.

For every resource use parameter, we obtained unit costs, if possible, using published Swiss sources or adjusting foreign sources to reflect Swiss practice. Drug unit costs for HCV treatment and treatment-related AEs were derived from the list of pharmaceutical specialties of the Swiss Federal Office of Public Health [40]. AE management costs were obtained by multiplying incidences of AEs such as anaemia, nausea, vomiting and rash with drug unit costs for their treatment. Unit costs for monitoring were obtained from Swiss tariff lists [41] or the analyses list of the Swiss Federal Office of Public Health [42]. If not otherwise indicated, unit costs were for the year 2014. Health state costs for the year 2012 were collected at the Gastroenterology and Hepatology clinic, University Hospital of Zurich. They thus represent a single centre experience. All cost parameters are shown in Table 3.

**Table 3:** Cost parameters and their ranges used in the deterministic and probabilistic sensitivity analyses.

	Item		Source	Base-case (CHF)	Sensitivity analysis	
*Treatment-related drug unit costs*	Drug	Cost/pack or injection		Cost/unit (CHF)	PSA distribution and parameters (DSA ranges)	PSA distribution and parameters (DSA ranges)
	Sofosbuvir, 28x400mg	19208.50	FOPH	1.72	Uniform, α = 14406 β = 24011	Uniform, α = 14406 β = 24011
	Ribavirin, 56x400mg	738.25	FOPH	0.03	Uniform, α = 664 β = 812	Uniform, α = 664 β = 812
	Peg. Interferon 2a, 180 μg	278.31	FOPH	1.55	Uniform, α = 250 β = 306	Uniform, α = 250 β = 306
	Peg. Interferon 2b, 120 μg	314.84	FOPH	2.62	Uniform, α = 283 β = 346	Uniform, α = 283 β = 346
	Telaprevir, 42x375mg	2948.70	FOPH	0.019	Uniform, α = 2654 β = 3244	Uniform, α = 2654 β = 3244
	Boceprevir, 336x200mg	4163.75	FOPH	0.06	Uniform, α = 3747 β = 4580	Uniform, α = 3747 β = 4580
*Treatment-related adverse event costs*	Item	Dosage	Source	Unit cost	Lower -25%	Upper +25%
	AE costs		[43]*	8938.40	6703.80	11173.00
	Nausea: 4 wks Metoclopramide	30mg/day	FOPH	0.019	-	-
	Vomiting: 4 wks Metoclopramide	30mg/day	FOPH	0.019	-	-
	Diarrhoea: 4.3 wks Loperamide	2mg/day	FOPH	0.241	-	-
	Pruritus: 4wks Piriton	16mg/day	FOPH	0.200	-	-
	Anaemia: 4 wks Binocrit	40,000/ wk	FOPH	0.010	-	-
	Anaemia: blood transfusion		FOPH	3,325.00	-	-
	Rash: 4 wks Hydrocortisone	1% 15g	FOPH	7.90	-	-
	Thrombocytopenia: 4 wks Revolade	50mg/day	FOPH	1.94	-	-
	Neutropenia: 2 wks Filgrastim	5 μg/kg/ day	FOPH	0.999	-	-
	Depression: 4 wks Citalopram	20mg/day	FOPH	0.064	-	-
*Treatment-related monitoring unit costs*	Item		Source	Unit cost	-	-
	Nurse	1 hour	Spitex	79.80	-	-
	Physician	1 hour	Tarmed	181.77	-	-
	Inpatient care	1 hour	Tarmed	23.71	-	-
	HCV screen (RNA), viral load, genotype, SVR test, HIV RNA		FOPH	180.00	-	-
	HBV, Anti-HIV		FOPH	20.00	-	-
	Liver function test		FOPH	5.00	-	-
	Alfa-fetoprotein		FOPH	19.30	-	-
	Alfa-antitrypsin		FOPH	23.00	-	-
	Thyrotrophic, Free T4		FOPH	9.00	-	-
	Caeruloplasmin		FOPH	19.90	-	-
	Iron		FOPH	2.80	-	-
	Urea and electrolytes		FOPH	11.20	-	-
	Glucose, Alanine aminotransferase		FOPH	2.50	-	-
	Pregnancy test		FOPH	12.00	-	-
	Thyroid function tests		FOPH	18.00	-	-
	Full blood count, blood clotting factors		FOPH	4.20	-	-
	Ferritin		FOPH	7.90	-	-
	Blood group		FOPH	17.10	-	-
	Autoantibodies		FOPH	37.00	-	-
	Immunoglobulins		FOPH	6.20	-	-
	Ultrasound scan of liver		Tarmed	113.69	-	-
	Chest X-ray		Tarmed	75.62	-	-
	Ultrasound guided biopsy		Tarmed	179.05	-	-
	Ultrasound of liver. Fibroscan		Tarmed	77.77	-	-
	Electrocardiography		Tarmed	55.67	-	-
	Magnetic resonance imaging of liver		Tarmed	390.14	-	-
	Pulmonary function test		Tarmed	62.15	-	-
	Liver biopsy		Tarmed	255.95	-	-
	Fibrotest		FOPH	69.10	-	-
	Endoscopy diagnosis		Tarmed	337.90	-	-
*Health state costs*	Item		Source	Base-case (CHF)	DSA ranges (CHF)	PSA distribution and parameters
	Non-cirrhotic		Calc.#	479	64–1301	Gamma, α = 2 β = 224
	Non-cirrhotic, mild		Expert opinion§	283	-	-
	Non-cirrhotic, moderate		Expert opinion§	1138	-	-
	Non-cirrhotic, SVR		Calc.#	366	275–458	Gamma, α = 8 β = 47
	Non-cirrhotic, mild, SVR		[31]&	348	-	-
	Non-cirrhotic, moderate, SVR		[31]&	426	-	-
	Compensated cirrhosis		Expert opinion§	2,715	1,357–4,535	Gamma, α = 11 β = 246
	Compensated cirrhosis, SVR		[31]&	754	282–779	Gamma, α = 5 β = 161
	Decompensated cirrhosis		Expert opinion§	20,347	16,561–24,517	Gamma, α = 13 β = 1510
	Hepatocellular carcinoma		Expert opinion§	16,944	6,163–33,082	Gamma, α = 6 β = 2865
	Liver transplant		Expert opinion§	125,102	93,827–156,378	-
	Post-liver transplant		Expert opinion§	19,323	14,492–24,154	Gamma, α = 4 β = 4471

AE, adverse event; Calc., calculation; FOPH, Federal Office of Public Health; GP, general practitioner; HBV, hepatitis B virus; HCV, hepatitis C virus; HIV, human immunodeficiency virus; mg, milligram; Peg., pegylated; wks, weeks; RNA, Ribonucleic acid; SOF, Sofosbuvir; SVR, sustained virological response; TE, treatment-experienced; wks, weeks

* converted from 2012 US Dollars to 2012 Swiss francs by using [44–46]

# weighted average: 77% mild, 23% moderate

§ provided by one of the authors (BM), Gastroenterology and Hepatology clinic, University Hospital of Zurich

& inflated by 1.14 to £ 2012 and converted to Swiss Francs (exchange rate 1.52)

### Subgroup analyses

In subgroup analyses, the cost-effectiveness of sofosbuvir was calculated separately for non-cirrhotic (fibrosis stage 0–3) and cirrhotic patients (fibrosis stage 4) based on the METAVIR classification [23].

### Sensitivity analyses

We performed extensive deterministic (DSA) and probabilistic sensitivity analysis (PSA) to explore the impact of parameter uncertainty on the cost-effectiveness results. The ranges of variation for DSA and the distributional assumptions and parameters used for PSA are listed in Tables 1 and 3. SVR rates were varied by ±25% of the base-case SVR rate.

To gain a better understanding of the impact of AEs on the cost-effectiveness of sofosbuvir-based strategies versus current standard of care, we performed a scenario analysis including total cost of AE management. The incidence of severe AEs was obtained from published clinical trials (Table 2). Costs of AE management were obtained from Bichoupan et al. [43]. The authors reported the total treatment costs for AE management in CHC patients treated with a combination of telaprevir, pegylated interferon alpha and ribavirin in the USA. We assumed that 80% of these total treatment costs were due to severe AEs. This cost parameter was adjusted for differences in the amount of services used, approximated by health care expenditure per head of the population [46]. Foreign prices were additionally adjusted using purchasing power parities [45]; prices were further adjusted to the increase in health expenditure using national statistical data [44].

## Results

### Base-case analysis

In the base-case analysis including a mixed population of about 25% cirrhotic and 75% non-cirrhotic CHC patients as seen in the SCCS, the incremental cost-effectiveness of therapy with sofosbuvir depended on genotype, treatment history, and interferon tolerability (Table 4). Sofosbuvir-containing treatment regimens in patients with CHC genotype 1–4 led to base-case ICERs between CHF 10,337 and CHF 91,570 per QALY gained. ICERs are below CHF 50,000 per QALY in treatment-naïve interferon eligible genotype 1 patients and in treatment-naïve interferon ineligible genotype 2 and 3 patients.

**Table 4:** Summary of base-case, sensitivity and subgroup analysis results.

Indication	Treatment and comparator strategies	Base-case (21–25% cirrhotic*) ICERs	DSA ranges	PSA	Subgroup (100% cirrhotic) ICERs	Subgroup (100% non-cirrhotic) ICERs
*GT1 TN IE*	SOF + PegIFN2a + RBV for 12 wks vs. PegIFN2a/2b + RBV for 48 wks	19.474	6,339–31,668	100% of ICERs<100,000	1,565	36,501
	SOF + PegIFN2a + RBV for 12 wks vs. TEL + PegIFN2a + RBV for 24/48 wks	10,337	SOF dominant-44,639	98.2% of ICERs<100,000	SOF dominant	28,608
	SOF + PegIFN2a + RBV for 12 wks vs. BOC + PegIFN2b + RBV for 28/48 wks	13,276	SOF dominant-35,975	100% of ICERs<100,000	963	26,579
*GT1 TN II*	SOF + RBV for 24 wks vs. NT	86,648	42,713–148,297	50.8% of ICERs<100,000	75,799	95,741
*GT2 TN IE*	SOF + RBV for 12 wks vs. PegIFN2a/2b + RBV for 24 wks	76,526	40,468–138,263	58.7% of ICERs<100,000	35,302	115,138
*GT2 TN II*	SOF + RBV for 12 wks vs. NT	10,471	1,080–19,273	100% of ICERs<100,000	SOF dominant	17,808
*GT3 TN IE*	SOF + RBV for 24 wks vs. PegIFN2a/2b + RBV for 24 wks	91,570	47,672–130,036	48.9% of ICERs<100,000	28,384	189,063
	SOF + PegIFN2a + RBV for 12 wks vs. PegIFN2a/2b + RBV for 24 wks	38,512	17,109–57,694	97.4% of ICERs<100,000	8,491	74,341
*GT3 TN II*	SOF + RBV for 24 wks vs. NT	34,826	14,649–53,365	99.5% of ICERs<100,000	17,275	46,900
*GT3 TE IE*	SOF + RBV for 24 wks vs. PegIFN2a/2b + RBV for 48 wks	74,805	38,237–112,762	65.8% of ICERs<100,000	90,653	76,064
	SOF + PegIFN2a + RBV for 12 wks vs. PegIFN2a/2b + RBV for 48 wks	16,235	4,314–26,946	99.7% of ICERs<100,000	4,382	24,957
*GT3 TE II*	SOF + RBV for 24 wks vs. NT	45,935	20,783–68,920	96.6% of ICERs<100,000	36,189	52,720
*GT4 TN*	SOF + PegIFN2a + RBV for 12 wks vs. PegIFN2a/2b + RBV for 48 wks	36,108	17,738–87,678	83.9% of ICERs<100,000	89,131	33,054

BOC, boceprevir; DSA, deterministic sensitivity analysis; GT, genotype; ICER, incremental cost-effectiveness ratio; IE, interferon eligible; II, interferon ineligible; PegIFN, pegylated interferon; PSA, probabilistic sensitivity analysis; RBV, ribavirin; SOF, sofosbuvir; TE, treatment-experienced; TEL, telaprevir; TN, treatment-naïve; wks, weeks

* percentage of cirrhotic patients depends on genotype: 24% in genotype 1, 21% in genotype 2, 25% in genotype 3 and 22% in genotype 4

ICERs for treatment-naïve genotype 1 and 4 patients receiving sofosbuvir, pegylated interferon alpha and ribavirin for 12 weeks ranged from CHF 10,337 to CHF 36,108 per QALY. The lowest ICERs for genotype 1 were obtained against telaprevir-containing regimens (CHF 10,337 per QALY) and boceprevir-containing regimens (CHF 13,276 per QALY). Underlying drug cost differences were smaller in these cases than in comparisons of sofosbuvir, pegylated interferon alpha and ribavirin with pegylated interferon alpha and ribavirin alone (Table 5). In treatment-naïve, interferon-ineligible genotype 1 patients, the treatment-related and total costs for a sofosbuvir-based treatment compared to no treatment were higher, as reflected in a higher ICER (CHF 86,648 per QALY).

**Table 5:** Cost (CHF) per patient and cost-effectiveness results in genotype 1.

Indication	Parameter	Intervention	Comparator	Δ	Comparator	Δ	Comparator	Δ	Comparator	Δ
*GT1 TN IE*		SOF+PaR 12 wks	PaR 48 wks		PbR 48 wks		TEL+PaR 48 wks		BOC+PbR 48 wks	
	Drug costs	62,862	20,454	42,408	20,875	41,987	49,333	13,529	45,135	17,727
	AE costs	15	2,064	-2,049	2,064	-2,049	3,086	-3,071	56	-41
	Monitoring costs	3,279	4,650	-1,371	4,650	-1,371	4,115	-836	4,404	-1,125
	Total treatment costs	66,156	27,168	38,988	27,589	38,567	56,534	9,622	49,595	16,561
	Health state costs	8,646	24,403	-15,757	24,398	-15,752	13,986	-5,340	16,897	-8,251
	Total cost per patient	74,802	51,571	23,231	51,987	22,815	70,520	4,282	66,492	8,310
	QALYs per patient*	13.3	12.1	1.2	12.1	1.2	12.9	0.4	12.7	0.6
*GT1 TN II*		SOF+RBV 24 wks	NT	**Δ**						
	Drug costs	120,184	0	120,184						
	AE costs	40	0	40						
	Monitoring costs	3,502	1,472	2,030						
	Total treatment costs	123,726	1,472	122,254						
	Health state costs	19,070	35,964	-16,894						
	Total cost per patient	142,796	37,436	105,360						
	QALYs per patient*	12.6	11.4	1.2						

AE, adverse event; BOC, boceprevir; GT, genotype; IE, interferon eligible; II, interferon ineligible; NT, no treatment; PaR, pegylated interferon alpha+ribavirin; PbR, pegylated interferon beta+ribavirin; QALY, quality-adjusted life year; RBV, ribavirin; SOF, sofosbuvir; TEL, telaprevir; TN, treatment-naïve

* values are rounded to one decimal place

In genotype 2, treatment-naïve patients we observed a substantial difference in the ICERs (Table 4) for interferon-eligible and interferon-ineligible patients (CHF 76,526 vs. CHF 10,471 per QALY). With a sofosbuvir-based treatment, interferon-ineligible patients gained 2.6 QALYs compared to no treatment, whereas interferon-eligible patients gained 0.6 QALYs compared to treatment with pegylated interferon alpha and ribavirin (S3 Table).

In genotype 3, ICERs of interferon-ineligible patients were in the same range for treatment-naïve (CHF 34,826 per QALY) and treatment-experienced (CHF 45,935 per QALY) patients (Table 4). The limited difference in ICERs was due to moderately lower QALY gains in treatment-experienced patients (2.1 vs. 2.5 QALY). We also noted a large difference in the cost per QALY gained of two different sofosbuvir-containing regimens, versus the same comparator, for the treatment of genotype 3, interferon-eligible patients (S4 Table). A treatment strategy of sofosbuvir in combination with pegylated interferon alpha and ribavirin for 12 weeks was clearly more cost effective than a 24 week-alternative without interferon in treatment-experienced (ICERs CHF 16,235 per QALY vs. CHF 74,805 per QALY, respectively) and treatment-naïve patients (ICERs CHF 38,512 per QALY vs. CHF 91,570 per QALY) (Table 4).

Total and disaggregated costs and QALYs gained per patient are presented in Table 5 for genotype 1. Cost and QALY results for the other HCV genotypes are presented in S3–S5 Tables. In genotype 1 treatment-naïve patients, drug costs for sofosbuvir-containing regimens were substantially higher than drug costs for comparator strategies (e.g. CHF 62,862 for sofosbuvir+pegylated interferon alpha+ribavirin vs. CHF 20,454 for pegylated interferon alpha+ribavirin). Monitoring costs were generally lower for sofosbuvir-containing regimens than comparator strategies (CHF 3,279 for sofosbuvir+ pegylated interferon alpha+ribavirin vs. CHF 4,650 for pegylated interferon alpha+ribavirin, respectively), except when the comparator strategy was no treatment. Health state costs were usually higher in comparator strategies than sofosbuvir-containing regimens (e.g. CHF 24,403 for pegylated interferon alpha+ribavirin vs. CHF 8,646 for sofosbuvir+ pegylated interferon alpha+ribavirin). QALYs per patient were always higher with treatment regimens including sofosbuvir, by 0.4 to 2.6 QALYs.

### Subgroup analysis

Subgroup-analyses were performed for cirrhotic and non-cirrhotic patients separately. Generally, costs per QALY gained were lower in cirrhotic patients than in non-cirrhotic patients (Table 4). In genotype 1 treatment-naïve, interferon-eligible patients with cirrhosis, sofosbuvir dominated (was clinically more advantageous and less expensive than) telaprevir- and boceprevir-containing treatment regimens. The cost per QALY was more than CHF 100,000 in genotypes 2 and 3 treatment-naïve, interferon-eligible, non-cirrhotic patients.

### Sensitivity analyses

The majority of the results of the DSA and PSA were robust and remained below CHF 100,000 per QALY gained (Table 4). Discount rates, the utility gain after treatment, and differences in SVR probabilities (SVR rates were varied by either ±20% of the base-case SVR rate or by estimating 95% confidence intervals assuming a beta distribution) were the most influential parameters. When we took into account an approximated total cost for severe AE management, ICERs of sofosbuvir-based treatment versus current standard care generally changed by less than 5% from the base-case ICERs. In genotype 1, treatment-naïve, interferon-eligible patients, AE management costs per patient for telaprevir-containing regimens (CHF 1,877) and boceprevir-containing regimens (CHF 1,162) were higher than AE management costs per patient for sofosbuvir-based treatment (CHF 89). Hence, the corresponding ICERs of sofosbuvir-based treatment versus telaprevir-containing regimens (CHF 13,425 per QALY) and boceprevir-containing regimens (CHF 11,633 per QALY) increased by 30% and decreased by 12% from base-case, respectively. The ICER for telaprevir-containing regimens increased, because the incidence of severe AEs was lower than the cumulative incidence of AEs such as anaemia, nausea, vomiting and rash used in the base-case analysis.

## Discussion

Although the initial costs of sofosbuvir-containing regimens were high, AE costs, monitoring costs and health state costs were generally lower than in active comparator strategies. Additionally, QALYs gained per patient were consistently higher in sofosbuvir-containing regimens. In the base-case representing a mixed cirrhotic and non-cirrhotic cohort, ICERs for sofosbuvir-containing treatment regimens were better than CHF 100,000 per QALY, for all comparisons. In the majority of sensitivity analyses, results were robust. ICERs of sofosbuvir-based regimens were also lower than CHF 100,000 per QALY in cirrhotic patients, but were above CHF 100,000 per QALY for treatment-naïve, interferon eligible genotype 2 and 3 non-cirrhotic patients.

SVR, the goal of antiviral therapy, can be achieved with a shorter duration of sofosbuvir-containing treatment than with other treatment regimens. For patients who are ineligible, intolerant or unwilling to take interferon-based regimens, the combination of sofosbuvir and ribavirin addresses an unmet medical need. There has been a shift in European and American guidelines in the way that EASL [20] and the American Association for the Study of Liver Diseases (AASLD/IDSA/IAS-USA) [47] no longer recommend treatment with telaprevir or boceprevir in genotype 1 patients. Given the recent marketing authorisation of a combination pill of sofosbuvir and ledipasvir for the treatment of HCV genotype 1 patients in October 2014 by the Food and Drug Administration [48] and more recently by the European Medicines Agency [49], further guideline adaptations may follow.

In our modelling study, sofosbuvir-containing treatment regimens compared to standard regimens led to gains in QALYs. This is in contrast to the German Institute for Quality and Efficiency in Health Care's (IQWiG) conclusion [50] that “there is no data documenting an additional benefit of sofosbuvir-containing regimens compared to other treatment regimens because no appropriate analyses were provided by the pharmaceutical company”. IQWiG’s position was partially due to a lack of head-to-head trials and the use of mixed treatment comparisons. Heterogeneity between patient characteristics may limit the validity of mixed treatment comparisons [51]. As patient characteristics such as age and genotype were comparable between studies, we regard mixed treatment comparisons as an acceptable approach to integrate available evidence, in the present case.

Leleu et al. published a cost-effectiveness study of sofosbuvir in mono-infected and co-infected CHC patients in France [52]. The resulting ICERs were better than in our study. Leleu et al. used a lower utility decrement for patients during treatment with sofosbuvir than for patients during treatment with comparators [52]. We used the same utility decrement for all treatments. In the French study, the percentages of cirrhotic patients were up to twice as high as in the base-case of our study. We showed that ICERs for cirrhotic patients were better than ICERs for non-cirrhotic patients. Hence, these differences in model input parameters may explain the variation in the magnitude of the ICERs. Another cost-effectiveness study in France by Deuffic-Burban et al. [53] compared sofosbuvir-containing treatment regimens and standard treatments in genotype 1 treatment-naïve CHC patients. The authors concluded that treatment with sofosbuvir is cost-effective in CHC patients with fibrosis stage 2 or higher [53]. An Italian cost-effectiveness study by Petta et al. [54] compared triple therapies with sofosbuvir, boceprevir, and telaprevir. Sofosbuvir-based regimens were cost-effective compared to boceprevir, except in cirrhotic and *IL28B* CC patients, and mostly cost-effective compared to telaprevir. A third cost-effectiveness study in the US concluded that sofosbuvir-based treatment regimens generally dominated telaprevir or boceprevir-based regimens [55]. The authors of a Spanish cost-effectiveness study reported that treatment regimens with sofosbuvir, pegylated interferon alpha and ribavirin for 12 weeks were below the considered cost-effectiveness threshold [56]. Overall, the results of the international cost-effectiveness studies were similar to the present analysis. However, transferability of cost-effectiveness results between different countries is limited due to e.g. differences in epidemiology of the disease, clinical practice, consumer preferences and price levels [57].

In Switzerland, there is no formally accepted cost-effectiveness threshold. In 2010, the Swiss Federal Court of Justice decided that treating a very rare orphan disease at CHF 500,000 per year was too expensive for the compulsory health insurance to cover, given its small health benefits [58]. The Court further stated that beyond a threshold of CHF 100,000 per QALY, health insurers cannot be obliged to pay for a treatment [58]. This was the first time that a formal cost-effectiveness threshold has been suggested for Switzerland to distinguish favourable from unfavourable ICERs. On this basis, sofosbuvir-containing regimens would be cost-effective compared to standard treatment. However, this remains arguable and there will be further discussion, also given widely varying approaches in other countries. NICE's acceptable cost-effectiveness thresholds for the UK are in the range of GBP 20,000 to 30,000 per QALY gained and are intended to represent the opportunity cost to a fixed National Health Service budget [59]. The cost-effectiveness threshold commonly adopted in studies for the US varies between USD 50,000 to 100,000 per QALY [60]. The World Health Organization (WHO) suggests cost-effectiveness thresholds between 2–3 times the gross domestic product (GDP) per person [61]. The Swiss GDP per person was CHF 74,010 in 2012 [62]. According to the threshold suggested by WHO, all results including subgroup analyses would be cost-effective.

An additional point of discussion relates to the fact that thresholds representing the societal willingness to pay for any intervention leading to health gains may vary depending on the context. This was pointed out in a recent article that emphasized how the cost-effectiveness of sofosbuvir may dramatically clash with its affordability if the treatment is to be implemented on a large scale [63]. Not surprisingly, economic studies consider ICERs in distinct subgroups of patients. This appears palatable in those patients in whom the benefit is clear cut, i.e. patients with advanced liver fibrosis at high risk of complications, while it becomes increasingly difficult to accept by payers for patients in whom the life-long risk of complications is relatively small, i.e. patients with no or minimal fibrosis. Those patients are significantly less likely to die of HCV-related consequences and treatment-induced viral clearance may lead to less clear health gains.

Our study has several strengths and limitations relating to the decision-analytic model’s structural assumptions and available input data. Patient characteristics were obtained from the SCCS [19]. Swiss patient characteristics are thus reflected and the local applicability of the results is improved. The validity of the model was assessed by scrutinizing outputs for internal consistency. Model elements and formulae were thoroughly checked for correctness. We also compared the structure with that of other models in the field, which supported the approaches taken.

Clinical input parameters such as transition probabilities and utilities were derived from international literature. Transition probabilities reflect the biological course of CHC and mainly depend on disease characteristics and treatment. European countries with similar treatment options available should have comparable transition probabilities. The utilities used in our model were mainly based on UK studies [21,22,29]. Although the EQ-5D is a generic instrument to measure quality of life, the value sets applied to obtain utilities differ between countries [64]. The values available to us were based on the UK value set. There is no Swiss-specific value set, but a recent study reported that the UK value set generates lower utilities than the German or French value set [65]. Because of the small difference, we have no reason to assume that this had a substantial impact on our results, at least not in the sense of favouring sofosbuvir. For sofosbuvir and alternative treatment regimens we used the same utilities according to health state because treatment-specific utilities were not available. Given the lifelong time horizon of the model, this should not substantially impact the findings. Treatment characteristics and medical resource use based on clinical trial data, international literature and European guidelines were checked by Swiss clinical experts and generally found to be applicable to the Swiss situation. A small number of assumptions were, however, modified. For example, all HCV patients in Switzerland are tested for hepatitis B virus infection while there is no cryoglobulin test performed.

To the extent feasible, the model was populated with Swiss input parameters for patient characteristics, health state costs and other unit costs. This implied some uncertainties. For the patient characteristics implemented in the model, we used data from the SCCS, which reflects local patient characteristics and medical practice. However, patients in the clinical trials of sofosbuvir were younger than patients in the SCCS. If relevant SVR effects, adverse event rates, etc. were age-dependent, this age difference could distort the study results. In addition, we received health state costs from one big university hospital in Switzerland, which were based on a data collection of one year. These costs could differ from those accrued in other settings in Switzerland, which might lead to different results. Despite these aspects and because the model structure, underlying assumptions and model parameters were reviewed for appropriateness by Swiss clinical experts, we believe to have achieved reasonable estimates of the cost effectiveness of CHC treatment with sofosbuvir in Switzerland.

In the base-case analysis, we only included drug costs for the treatment of AEs due to a lack of Swiss specific data. In this respect, this modelling study may provide a conservative estimate of the cost-effectiveness of sofosbuvir-based HCV treatment regimens. In a scenario analysis, total costs of severe AE management were obtained from a US study [43] and adjusted to Swiss costs to the extent possible. ICERs in this scenario analysis changed by less than 5% in comparison with the base case, except for telaprevir-containing and boceprevir-containing regimens. Given differences in regimen-specific AE profiles, data allowing for the inclusion of total AE management costs (i.e. not only drug costs to treat AEs) might improve ICERs of sofosbuvir treatment.

Given the model structure and underlying model input parameters (mild and moderate HCV states (fibrosis stage F0-F3) were combined to a non-cirrhotic state to reflect available data from clinical trials), it was not possible to calculate ICERs for sofosbuvir-based regimens compared to standard treatment according to fibrosis stage. Therefore, the model is not able to reflect the current Swiss treatment limitations. In some cases, SVR rates were obtained from phase II trials due to missing or inapplicable data from phase III trials. Nevertheless, SVR rates from phase II and III trials were consistent. The cost-effectiveness for genotype 1 treatment-experienced could not be assessed due to lack of clinical data. As there are no data available regarding the reversibility of complications after treatment and any assumption made would add uncertainty to the model, we did not consider this and assumed a similar post-treatment clinical course independent from the type of treatment used. Clinical efficacy data were derived from registration trials and may not fully reflect effectiveness in routine clinical practice due to compliance and adherence issues, which limits the generalisability of the results. Finally, the analysis was conducted from the health care system perspective. A broader societal perspective including indirect costs (e.g. due to loss of productivity, absence from work) would provide additional insights into the implications of sofosbuvir-based therapy.

Several authors have criticised the high price of sofosbuvir [66–68]. Given a relevant pool of prevalent, previously untreated HCV cases, budget impact remains an issue among payers and policy makers, especially due to the high up-front costs. Swiss regulators restricted the reimbursement of sofosbuvir-containing treatment to patients with fibrosis stage 3 or 4, or symptomatic patients with extra-hepatic manifestations [17], which reduces initial budget impact. At the same time, our findings for Switzerland generally indicate acceptable ICERs for sofosbuvir-containing regimens, which may initiate further discussion on reimbursement issues. Our results further provide a benchmark for future treatment regimens in patients with CHC that are expected to become available in the near future.

## Supporting Information

S1 Table Specific adverse events. BOC, boceprevir; GT, genotype; IE, interferon eligible; II, interferon ineligible; NA, not available; PegIFN, pegylated interferon; RBV, ribavirin; SOF, sofosbuvir; SVR, sustained virological response; TE, treatment-experienced; TEL, telaprevir; TN, treatment-naïve; # 1% erythropoietin and 0.7% blood transfusions; § equally distributed between erythropoietin and blood transfusions.
(PDF)

S2 Table Resource use for monitoring based on EASL Clinical Practice and Treatment Guidelines. BOC, boceprevir; C, cirrhotic; HBV, hepatitis B virus; HCV, hepatitis C virus; HIV, human immunodeficiency virus; MRI, magnetic resonance imaging; NC, non-cirrhotic; PegIFN, pegylated interferon; RBV, ribavirin; RNA, ribonucleic acid; SOF, sofosbuvir; SVR, sustained virological response; TEL, telaprevir; *resource use was checked by Swiss clinical experts; 1 stands for 100% of the patients.
(PDF)

S3 Table Cost (CHF) per patient and cost-effectiveness results in genotype 2. AE, adverse event; IE, interferon eligible; II, interferon ineligible; NT, no treatment; PaR, pegylated interferon 2a+ribavirin; PbR, pegylated interferon 2b+ribavirin; QALY, quality-adjusted life year; RBV, ribavirin; SOF, sofosbuvir; TN, treatment-naïve; * values are rounded to one decimal place.
(PDF)

S4 Table Cost (CHF) per patient and cost-effectiveness results in genotype 3. AE, adverse event; IE, interferon eligible; II, interferon ineligible; NT, no treatment; PaR, pegylated interferon 2a+ribavirin; PbR, pegylated interferon 2b+ribavirin; QALY, quality-adjusted life year; RBV, ribavirin; SOF, sofosbuvir; TE, treatment-experienced; TN, treatment-naïve; * values are rounded to one decimal place.
(PDF)

S5 Table Cost (CHF) per patient and cost-effectiveness results in genotype 4. AE, adverse event; IE, interferon eligible; II, interferon ineligible; NT, no treatment; PaR, pegylated interferon 2a+ribavirin; PbR, pegylated interferon 2b+ribavirin; QALY, quality-adjusted life year; RBV, ribavirin; SOF, sofosbuvir; TE, treatment-experienced; TN, treatment-naïve; * values are rounded to one decimal place.
(PDF)
