# Supplementary material for: Cost-Effectiveness Analysis of Sofosbuvir Compared to Current Standard Treatment in Swiss Patients with Chronic Hepatitis C
Source: PLoS One. 2015 May 14;10(5):e0126984. doi: 10.1371/journal.pone.0126984 (PMC4431849; doi:10.1371/journal.pone.0126984)
Supplement: S1 Table — BOC, boceprevir; GT, genotype; IE, interferon eligible; II, interferon ineligible; NA, not available; PegIFN, pegylated interferon; RBV, ribavirin; SOF, sofosbuvir; SVR, sustained virological response; TE, treatment-experienced; TEL, telaprevir; TN, treatment-naïve; # 1% erythropoietin and 0.7% blood transfusions; § equally distributed between erythropoietin and blood transfusions. (PDF) [file pone.0126984.s001.pdf]

S1 Table. Specific adverse events

**Indication**<br>***Source***	**SOF-based and comparator strategies**	**Patients with adverse events**								
		Nausea	Vomiting	Pruritus	Rash	Anaemia	Thrombocytopenia	Neutropenia	Depression	CNS
GT 1 TN IE<br>[13, 57, 58]	SOF + PegIFN2a + RBV for 12 wks<br>PegIFN2a/2b + RBV for 48 wks<br>TEL + PegIFN2a + RBV for 24/48 wks<br>BOC + PegIFN2b + RBV for 24/48 wks	-<br>1%<br>1%<br>-	-	-<br>0.1%<br>1.3%<br>-	-<br>0.1%<br>4.8%<br>-	1.7%#<br>49.8%#<br>99.6%§<br>1.6%§	-<br>0.6%<br>2.5%<br>-	-<br>14.7%<br>10.3%<br>0.3%	-<br>0.3%<br>-<br>0.3%	-<br>0.9%<br>0.5%<br>1%
GT 1 TN II<br>[59]	SOF + RBV for 24 wks<br>NT	0.8%<br>-	0.4%<br>-	-	-	1.7%#<br>-	-	-	-	-
GT 2 TN IE<br>[13]*	SOF + RBV for 12 wks<br>PegIFN2a/2b + RBV for 24 wks	-	-	-	-	1.2%§<br>-	-	-	-	-
GT 2 TN II<br>[12]	SOF + RBV for 12 wks<br>NT	-	-	-	-	0.6%§<br>-	-	-	-	-
GT 3 TN IE<br>[13, 16, 60, 61]	SOF + RBV for 24 wks<br>SOF + PegIFN2a + RBV for 12 wks<br>PegIFN2a/2b + RBV for 24 wks	0.8%<br>-<br>-	0.4%<br>-<br>-	-	-	1.6%§<br>0.6%§<br>-	-	-	-	-
GT 3 TN II<br>[60]	SOF + RBV for 24 wks<br>NT	0.8%<br>-	0.4%<br>-	-	-	1.6%§<br>-	-	-	-	-
GT 3 TE IE<br>[16, 60, 62, 63]	SOF + RBV for 24 wks<br>SOF + PegIFN2a + RBV for 12 wks<br>PegIFN2a/2b + RBV for 48 wks	0.8%<br>-<br>-	0.4%<br>-<br>-	-	-	1.6%§<br>0.6%§<br>-	-	-	-	-
GT 3 TE II<br>[60]	SOF + RBV for 24 wks<br>NT	0.8%<br>-	0.4%<br>-	-	-	1.6%§<br>-	-	-	-	-
GT 4 TN<br>[13]	SOF + PegIFN2a + RBV for 12 wks<br>PegIFN2a/2b + RBV for 48 wks	-	-	-	-	0.6%*<br>-	-	-	-	-

BOC, boceprevir; GT, genotype; IE, interferon eligible; II, interferon ineligible; NA, not available; PegIFN, pegylated interferon; RBV, ribavirin; SOF, sofosbuvir; SVR, sustained virological response; TE, treatment-experienced; TEL, telaprevir; TN, treatment-naïve

1% erythropoietin and 0.7% blood transfusions
§ equally distributed between erythropoietin and blood transfusions
