# Supplementary material for: Cost-Effectiveness Analysis of Sofosbuvir Compared to Current Standard Treatment in Swiss Patients with Chronic Hepatitis C
Source: PLoS One. 2015 May 14;10(5):e0126984. doi: 10.1371/journal.pone.0126984 (PMC4431849; doi:10.1371/journal.pone.0126984)
Supplement: S2 Table — BOC, boceprevir; C, cirrhotic; HBV, hepatitis B virus; HCV, hepatitis C virus; HIV, human immunodeficiency virus; MRI, magnetic resonance imaging; NC, non-cirrhotic; PegIFN, pegylated interferon; RBV, ribavirin; RNA, ribonucleic acid; SOF, sofosbuvir; SVR, sustained virological response; TEL, telaprevir; *resource use was checked by Swiss clinical experts; 1 stands for 100% of the patients. (PDF) [file pone.0126984.s002.pdf]

S2 Table. Resource use for monitoring based on EASL Clinical Practice and Treatment Guidelines

Item	Details	Resource use*
Initial evaluation of a new patient with confirmed HCV before starting treatment (for all treatments)		
Gastroenterology	Consultant-led outpatient visit, HCV screen (RNA), SVR test, HIV RNA, Anti-HIV, liver function test, alfa-fetoprotein(cirrhotic patients), alfa-antitrypsin, thyrotropin, free T4, caeruloplasmin, iron	1
Tests and investigations	Urea and electrolytes, glucose, full blood count, ferritin, blood clotting factors, autoantibodies, immunoglobulins, ultrasound scan of liver, HCV genotype, endoscopy, HBV testing	1
	MRI liver (cirrhotic patients)	0.15
	Pulmonary function test (5% of patients)	0.05
Further investigations before starting treatment for every treatment group (SOF, PegIFN+RBV, BOC, TEL)		
Gastroenterology	Consultant-led outpatient vist	1
Inpatient care	Day case (half an hour)	0.5
Tests and investigations	Pregnancy test (5% of patients)	0.05
	Full blood count, blood clotting factors, blood group, HCV quantitative PCR	1
	Ultrasound guided biopsy, liver biopsy (in pathology)	0.2
	Fibroscan/Fibrotest	0.8

Monitoring during certain duration of sofosbuvir treatment (number of visits or tests during weeks of treatment)					
Details	4 wks	8 wks	12 wks	16 wks	24 wks
Consultant-led outpatient visit	3	4	5 (+1)	6 (+1)	6 (+1)
HCV screen (RNA), SVR test	2	2	2 (+1)	2 (+1)	2 (+1)
Liver function test	3	4	5 (+1)	6 (+1)	6 (+1)
Alfa-fetoprotein (cirrhotic only)	-	-	1	1	1
Urea and electrolytes	3	4	5 (+1)	6 (+1)	6 (+1)
Full blood count	3	4	5 (+1)	6 (+1)	6 (+1)
Thyroid function test	-	-	1	1	1
Blood clotting factors	2	2	2 (+1)	2 (+1)	2 (+1)

Monitoring during certain duration of comparator treatment (number of visits or tests during weeks of treatment)												
Details	4 wks		8 wks		12 wks		16 wks		24 wks		48 wks	
	C	NC	C	NC	C	NC	C	NC	C	NC	C	NC
Consultant-led outpatient visit	4	4	6	6	8	7	9	8	12	10	22	16
HCV screen (RNA), SVR test	1	1	1	1	1	1	1	1	2	2	2	2
Liver function test	4	4	6	6	8	7	9	8	12	10	22	16
Alfa-fetoprotein	-	-	-	-	1	-	1	-	2	1	2	1
Urea and electrolytes	4	4	6	6	8	7	9	8	12	10	22	16
Thyroid function test	-	-	-	-	1	1	1	1	2	2	4	4
Full blood count	4	4	6	6	8	7	9	8	12	10	22	16
Blood clotting factors	2	2	2	2	2	2	2	2	3	3	5	5
Ultrasound scan of liver	-	-	-	-	-	-	-	-	-	-	1	-
Endoscopy	-	-	-	-	-	-	-	-	-	-	1	-

BOC, boceprevir; C, cirrhotic; HBV, hepatitis B virus; HCV, hepatitis C virus; HIV, human immunodeficiency virus; MRI, magnetic resonance imaging; NC, non-cirrhotic; PegIFN, pegylated interferon; RBV, ribavirin; RNA, ribonucleic acid; SOF, sofosbuvir; SVR, sustained virological response; TEL, telaprevir

*resource use was checked by Swiss clinical experts; 1 stands for 100% of the patients
