# Supplementary material for: Cost-Effectiveness Analysis of Sofosbuvir Compared to Current Standard Treatment in Swiss Patients with Chronic Hepatitis C
Source: PLoS One. 2015 May 14;10(5):e0126984. doi: 10.1371/journal.pone.0126984 (PMC4431849; doi:10.1371/journal.pone.0126984)
Supplement: S3 Table — AE, adverse event; IE, interferon eligible; II, interferon ineligible; NT, no treatment; PaR, pegylated interferon 2a+ribavirin; PbR, pegylated interferon 2b+ribavirin; QALY, quality-adjusted life year; RBV, ribavirin; SOF, sofosbuvir; TN, treatment-naïve; * values are rounded to one decimal place. (PDF) [file pone.0126984.s003.pdf]

S3 Table. Cost (CHF) per patient and cost-effectiveness results in genotype 2

Indication<br>Parameter	Intervention	Comparator 1		Comparator 2	
	**SOF+RBV 12 wks**	**PaR 24 wks**	**Δ**	**PbR 24 wks**	**Δ**
Genotype 2 TN IE					
Drug costs	60,394	10,200	50,194	10,450	49,944
AE costs	29	0	29	0	29
Monitoring costs	3,295	3,799	-504	3,799	-504
Total treatment costs	63,718	13,999	49,719	14,249	49,469
Health state costs	7,323	14,380	-7,057	14,379	-7,056
Total costs per patient	71,041	28,379	42,662	28,628	42,413
QALYs per patient*	13.8	13.2	0.6	13.2	0.6
	**SOF+RBV 12 wks**	**NT**	**Δ**		
Genotype 2 TN II					
Drug costs	59,760	0	59,760		
AE costs	17	0	17		
Monitoring costs	3,277	1,472	1,805		
Total treatment costs	63,054	1,472	61,582		
Health state costs	6,480	41,362	-34,882		
Total costs	69,534	42,834	26,700		
QALYs per patient*	13.8	11.2	2.6		

AE, adverse event; IE, interferon eligible; II, interferon ineligible; NT, no treatment; PaR, pegylated interferon 2a+ribavirin; PbR, pegylated interferon 2b+ribavirin; QALY, quality-adjusted life year; RBV, ribavirin; SOF, sofosbuvir; TE, treatment-experienced; TN, treatment-naïve

* values are rounded to one decimal place
