# Supplementary material for: Cost-Effectiveness Analysis of Sofosbuvir Compared to Current Standard Treatment in Swiss Patients with Chronic Hepatitis C
Source: PLoS One. 2015 May 14;10(5):e0126984. doi: 10.1371/journal.pone.0126984 (PMC4431849; doi:10.1371/journal.pone.0126984)
Supplement: S4 Table — AE, adverse event; IE, interferon eligible; II, interferon ineligible; NT, no treatment; PaR, pegylated interferon 2a+ribavirin; PbR, pegylated interferon 2b+ribavirin; QALY, quality-adjusted life year; RBV, ribavirin; SOF, sofosbuvir; TE, treatment-experienced; TN, treatment-naïve; * values are rounded to one decimal place. (PDF) [file pone.0126984.s004.pdf]

S4 Table. Cost (CHF) per patient and cost-effectiveness results in genotype 3

Indication	Intervention	Comparator 1		Comparator 2	
**Parameter**	**SOF+RBV 24 wks**	**PaR 24 wks**	**Δ**	**PbR 24 wks**	**Δ**
Genotype 3 TN IE					
Drug costs	120,590	9,720	110,870	9,958	110,632
AE costs	40	0	40	0	40
Monitoring costs	3,486	3,735	-249	3,736	-250
Total treatment costs	124,116	13,455	110,661	13,694	110,422
Health state costs	6,282	21,763	-15,391	21,762	-15,480
Total costs	130,398	35,218	95,180	35,456	94,942
QALYs per patient*	13.8	12.7	1.1	12.7	1.1
	**SOF+PaR 12 wks**	**PaR 24 wks**	**Δ**	**PbR 24 wks**	**Δ**
Genotype 3 TN IE					
Drug costs	63,734	9,720	54,014	9,958	53,776
AE costs	15	0	15	0	15
Monitoring costs	2,805	3,735	-930	3,736	-931
Total treatment costs	66,554	13,455	53,099	13,694	52,860
Health state costs	7,572	21,763	-14,191	21,762	-14,190
Total costs	74,126	35,218	38,908	35,456	38,670
QALYs per patient*	13.8	12.7	1.1	12.7	1.1
	**SOF+RBV 24 wks**	**NT**	**Δ**		
Genotype 3 TN II					
Drug costs	120,590	0	120,590		
AE costs	40	0	40		
Monitoring costs	3,486	1,472	2,014		
Total treatment costs	124,116	1,472	122,644		
Health state costs	6,282	41,362	-35,080		
Total costs	130,398	42,834	87,564		
QALYs per patient*	13.7	11.2	2.5		
	**SOF+RBV 24 wks**	**PaR 48 wks**	**Δ**	**PbR 48 wks**	**Δ**
Genotype 3 TE IE					
Drug costs	120,590	18,125	102,465	18,569	102,021
AE costs	40	0	40	0	40
Monitoring costs	2,202	3,402	-1,200	3,402	-1,200
Total treatment costs	122,832	21,527	101,305	21,971	100,861
Health state costs	13,425	27,342	-13,917	27,337	-13,912
Total costs	136,257	48,869	87,388	49,308	86,949
QALYs per patient*	13.3	12.1	1.2	12.1	1.2
	**SOF+PaR 12 wks**	**PaR 48 wks**	**Δ**	**PbR 48 wks**	**Δ**
Genotype 3 TE IE					
Drug costs	58,865	18,125	40,740	18,569	40,296
AE costs	15	0	15	0	15
Monitoring costs	1,441	3,402	-1,961	3,402	-1,961
Total treatment costs	60,321	21,527	38,794	21,971	38,350
Health state costs	10,509	27,342	-16,833	27,337	-16,828
Total costs	70,830	48,869	21,961	49,308	21,522
QALYs per patient*	13.5	12.1	1.4	12.1	1.4
	**SOF+RBV 24 wks**	**NT**	**Δ**		
Genotype 3 TE II					
Drug costs	120,590	0	120,590		
AE costs	40	0	40		
Monitoring costs	2,025	189	1,836		

Total treatment costs	122,655	189	122,466		
Health state costs	13,425	41,366	-27,941		
Total costs	136,080	41,555	94,525		
QALYs per patient*	13.3	11.2	2.1		

AE, adverse event; IE, interferon eligible; II, interferon ineligible; NT, no treatment; PaR, pegylated interferon 2a+ribavirin; PbR, pegylated interferon 2b+ribavirin; QALY, quality-adjusted life year; RBV, ribavirin; SOF, sofosbuvir; TE, treatment-experienced; TN, treatment-naïve

* values are rounded to one decimal place
