# Supplementary material for: Cost-Effectiveness Analysis of Sofosbuvir Compared to Current Standard Treatment in Swiss Patients with Chronic Hepatitis C
Source: PLoS One. 2015 May 14;10(5):e0126984. doi: 10.1371/journal.pone.0126984 (PMC4431849; doi:10.1371/journal.pone.0126984)
Supplement: S5 Table — AE, adverse event; IE, interferon eligible; II, interferon ineligible; NT, no treatment; PaR, pegylated interferon 2a+ribavirin; PbR, pegylated interferon 2b+ribavirin; QALY, quality-adjusted life year; RBV, ribavirin; SOF, sofosbuvir; TE, treatment-experienced; TN, treatment-naïve; * values are rounded to one decimal place. (PDF) [file pone.0126984.s005.pdf]

**S5 Table. Cost (CHF) per patient and cost-effectiveness results in genotype 4**

| **Indication** | **Intervention** | **Comparator 1** | | **Comparator 2** | |
|---|---|---|---|---|---|
| **Parameter** | **SOF+PaR 12 wks** | **PaR 48 wks** | **Δ** | **PbR 48 wks** | **Δ** |
| Genotype 4 TN | | | | | |
| Drug costs | 63,734 | 20,481 | 43,253 | 20,931 | 42,803 |
| AE costs | 15 | 0 | 15 | 0 | 15 |
| Monitoring costs | 3,286 | 2,797 | 489 | 2,797 | 489 |
| Total treatment costs | 67,035 | 23,278 | 43,757 | 23,728 | 43,307 |
| Health state costs | 12,050 | 21,633 | -9,583 | 21,628 | -9,578 |
| Total costs | 79,085 | 44,911 | 34,174 | 45,356 | 33,729 |
| QALYs per patient* | 14.1 | 13.2 | 0.9 | 13.2 | 0.9 |

AE, adverse event; IE, interferon eligible; II, interferon ineligible; NT, no treatment; PaR, pegylated interferon 2a+ribavirin; PbR, pegylated interferon 2b+ribavirin; QALY, quality-adjusted life year; SOF, sofosbuvir; TE, treatment-experienced; TN, treatment-naïve

* values are rounded to one decimal place
